# Understanding meaningful work in the context of technostress, COVID-19, frustration, and corporate social responsibility

**DOI:** 10.1177/00187267221139776

**Published:** 2023-01-07

**Authors:** Darija Aleksić, Matej Černe, Saša Batistič

**Affiliations:** University of Ljubljana, Slovenia, darija.aleksic@ef.uni-lj.si; University of Ljubljana, Slovenia, matej.cerne@ef.uni-lj.si; Tilburg University, Netherlandsm, S.Batistic@tilburguniversity.edu

**Keywords:** corporate social responsibility, COVID-19, frustration, meaningful work, techno-invasion

## Abstract

COVID-19 and digitalization represent important sources of many employees’ frustrations. In this article, we address the question of how employees can achieve meaningful work in such a challenging and frustrating context. Specifically, we investigate whether employees’ negative experiences related to technology use—that is, techno-invasion—leads to frustration and in turn reduces employee perceptions of meaningful work. In addition, we examine corporate social responsibility as a potential remedy that could mitigate these negative effects. The results of our four-wave longitudinal study of 198 working professionals collected during the first wave of the COVID-19 pandemic did not find support for a proposed negative direct effect of techno-invasion on meaningful work. However, we did find support that perceived corporate social responsibility moderates the indirect relationship between techno-invasion and meaningful work, mediated by frustration: for low levels of corporate social responsibility, techno-invasion results in higher levels of frustration, in turn reducing meaningful work. High levels of corporate social responsibility buffer this negative indirect effect. Implications for research and practice dealing with digitalization, meaningful work, and corporate social responsibility are discussed.

He who has a why to live for can bear almost any how. (Friedrich Nietzsche)

## Introduction

Many philosophers since ancient Greece have contemplated the meaning of life, a debate that is gaining new momentum in a modern society, which is characterized by an abundance of opportunities (Guinness, 2018). As most adults spend the majority of their waking hours at work, contemporary discourse on a meaningful life increasingly emphasizes the importance of meaningful work. It denotes work that is significant, worthwhile, and has positive meaning and purpose for the individual (Lysova et al., 2019; Rosso et al., 2010; Smids et al., 2020).

Research suggests that meaningful work is related to many positive organizationally relevant outcomes, such as work engagement, job satisfaction, commitment, withdrawal intentions, and self-rated job performance (see Allan et al., 2019 for meta-analytic evidence). Given these benefits to employees and their organizations, scholars share a strong interest in understanding the factors that promote meaningful work (Michaelson et al., 2014). Despite many valuable efforts conducted to examine individual, job, organizational, and societal factors of meaningful work, understanding how these factors are interrelated and how organizations can thus promote the meaningful work experience for their employees remains limited (Lysova et al., 2019). Moreover, meaningful work is created through a highly social and contextualized process of conditions and constraints (Wrzesniewski et al., 2003), and the existing literature points to the need to better understand how the broader political, social, and institutional context shapes meaningful work (Bailey et al., 2019).

In recent years, the digitalization context has significantly shaped employees’ meaning-making process at work (i.e., the process through which meaning of work is created or destroyed). Although digitalization has greatly influenced the value system (Nikitenko, 2019) and has brought many important advances to work and everyday life, the current trends’ and developments’ impact resulting from the digitalization of work on employees’ meaningful work is still under-researched (Symon and Whiting, 2019). The few studies that have addressed this topic (e.g., Smids et al., 2020; Lent, 2018) have theorized on how digitalization and meaningful work are related; however, they have not empirically tested the proposed assumptions, or delved into individuals’ specific technology-related experiences of employees, mechanisms, and boundary conditions that enable meaningful work to occur despite digitally constraining phenomena. In addition, the recent COVID-19^1^ pandemic has magnified digitalization trends (see Molino et al., 2020) and brought new challenges, which have greatly influenced employees’ perceptions of meaningful work.

We build on Lips-Wiersma and Morris’s (2009) holistic model of meaningful work and the organizational frustration model (Fox and Spector, 1999; Spector, 1978) to explain how the context of digitalization and the COVID-19 have shaped the meaning-making process about work. The primary tenet of the organizational frustration model is that there is a relationship between the “sources of frustration in organizations, and effects on organizations through the reactions of individuals” (Spector, 1978: 818). In the current study, we propose technostress, defined as “any negative impact on attitudes, thoughts, behaviors, or body psychology caused directly or indirectly by technology” (Weil and Rosen, 1997: 5), focusing specifically on the techno-invasion dimension, as a novel source of frustration in organizations that has been made further prevalent owing to the COVID-19 context. Consistent with the organizational frustration model, we argue that techno-invasion elicits emotional responses of frustration, a key concept around which our research model theoretically revolves, and that experienced frustration influences employees’ perceptions of meaningful work. We draw on Lips-Wiersma and Morris’s (2009) holistic model of meaningful work, which emphasizes that seeking the balance between the needs of *self* and *others* are inherent in the process of meaning-making process, to propose a novel mechanism for mitigating the negative effects of frustration on meaningful work. Specifically, we argue that perceived corporate social responsibility (CSR) can be considered as a source of meaning derived from contributing to others that encourages employees to meet their own needs as well, thereby influencing the relationship between technostress-induced frustration and employees’ perceptions of meaningful work.

This study offers several contributions to research on meaningful work and organizational frustration. A recent review has shown that while perceptions of meaningful work also depend on the overall social context, our understanding of how individual, organizational, and societal factors interact to facilitate meaningful work remains limited (Lysova et al., 2019). Therefore, our first contribution is aimed at advancing the growing body of literature on meaningful work by conceptualizing a comprehensive theoretical framework that explains how employees acknowledge meaningful work in circumstances that offer limited opportunities for meaning (e.g., in the context of the COVID-19 crisis). By focusing on individual-level phenomena arising from the digital context, we respond to calls for more research that not only considers the factors that promote or hinder meaningful work, but also advancing this line of inquiry into how the broader context shapes meaningful work (Bailey et al., 2019; Lysova et al., 2019; Mitra and Buzzanell, 2017). As digitalization is continuously changing the nature of work and there are no indicators of a trend in the opposite direction, digitalization and its potential downsides, which the COVID-19 pandemic further exacerbated, are also significantly shaping the future of work through their impact on meaningful work. By examining the relationship between techno-invasion, employee frustration, and meaningful work, we respond to the call to explore the negative aspects (i.e., the “dark side”) of employees’ digitalization experiences (Wood et al., 2019) and extend the existing literature on meaningful work by empirically investigating, for the first time, the influence of novel individual-level negative phenomena (i.e., techno-invasion and frustration) and feelings on meaningful work in a digital and challenging context.

Furthermore, by examining the moderating role of CSR in the indirect relationship between techno-invasion and meaningful work, mediated by frustration, we complement current research on meaningful work and organizational frustration by highlighting the critical importance of CSR as a source of meaning that counterbalances the source of frustration source and emotional response, thereby promoting meaningful work. Perceptions of CSR have the potential to mitigate the negative effects of frustration caused by techno-invasion causes, thereby facilitating meaningful work in adverse and stressful situations. By demonstrating that CSR promotes meaningful work under specific conditions arising from the digital and crisis context, we address the call to explore the conditions under which CSR can simultaneously lead to win–win outcomes in terms of business value and employee well-being (Aguinis and Glavas, 2019), advancing the discourse on how CSR reduces the feeling of frustration and thereby increases the possibility that employees find their work meaningful.

Second, we aim to contribute to the literature on organizational frustration by exploring how situational events arising from digitalization and the COVID-19 context affect the three elements of the organizational frustration model. In doing so, we integrate the holistic model of meaningful work with the organizational frustration model into a comprehensive framework. This juxtaposition allowed us to theorize and explore the negative influence of the source of frustration and emotional responses on the perception of meaningful work, as well as the mitigating role of serving others. Building on the idea that situational factors are related to a specific source of frustration (Bessière et al., 2006), we propose a novel source of frustration (i.e., techno-invasion) that elicits a frustration emotional response (i.e., frustration), leading to a novel frustration outcome (i.e., meaningful work), as predicted by Fox–Spector model of organizational frustration. We further propose that CSR positively influences the relationship between frustration emotional response and outcome. In contrast to previous research that has examined how meaningful work can mitigate the negative effects of frustration (e.g., Ugwu and Onyishi, 2018), we aim to advance the existing literature on organizational frustration by providing an integrative theoretical framework that explains how specific events and perceptions affect the elements of the organizational frustration model and thereby influence meaningful work. Finally, a potential contextual and empirical contribution can be seen in testing the proposed model using a four-wave longitudinal study conducted during the first wave of the COVID-19 pandemic. Thus, we advance research on the experience of meaningful work under these unique and stressful conditions.

The remainder of this article is structured as follows. We first present the theoretical background with the hypotheses development section in which we state the logic behind our research model and conceptualize hypotheses. An empirical section follows this in which we present our longitudinal analyses’ methods and results. We conclude by discussing the theoretical contributions, practical implications, and limitations with future directions stemming from our findings.

## Theoretical background and hypotheses development

**Figure 1.**
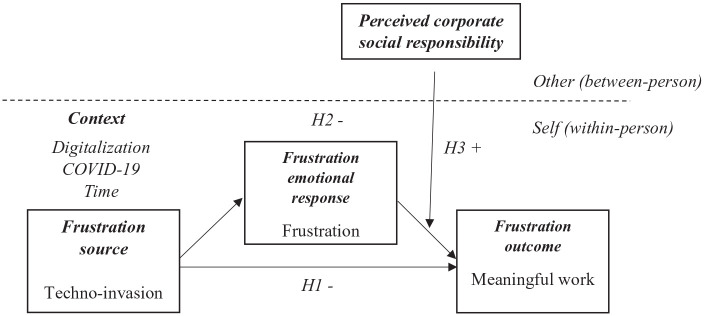
Integrated theoretical framework with hypotheses. Note. The integrated model explaining the logic behind our research is based on the juxtaposition of Lips-Wiersma and Morris’s holistic model of meaningful work (2009) and the Fox–Spector model of organizational frustration (Fox and Spector, 1999; Spector, 1978). ***Definitions of core constructs:*(1) Meaningful work**: work that is experienced as particularly significant and has a more positive meaning for the individual (Rosso et al., 2010), or more broadly, as “work that is personally significant and worthwhile” (Lysova et al., 2019: 375). **(2) Techno-invasion:** dimension of technostress referring to being constantly connected and thereby invading the employee’s personal life (Tarafdar et al., 2007). **(3) Frustration**: the feeling of being upset or annoyed as a result of being unable to change or achieve something (Boyd, 1982). **(4) Perceived corporate social responsibility:** refers to the degree of employees’ perception about the support their employer provides to the CSR-related activities (Choi and Yu, 2014).

Existing research indicates that stressful events can violate an individual’s perception of meaning of work and initiate the meaning-making process (Park, 2010; Park and George, 2013). We argue that stressful events arising from the context that digitalization and COVID-19 shaped can cause a crisis of meaning (i.e., evaluating life as frustratingly empty and lacking meaning). This study is grounded in the organizational frustration model (Fox and Spector, 1999; Spector, 1978) and holistic model of meaningful work (Lips-Wiersma and Morris, 2009) to explain how the digitalization and the COVID-19 context influenced employees perceptions of meaningful work and what are the mechanisms to cultivate the feeling of meaningful work in stressful conditions. The integrated theoretical framework presenting the research logic behind our model is displayed in Figure 1.

The organizational frustration model (Fox and Spector, 1999; Spector, 1978) specifies the relationships among sources of frustration, their effects on employees’ emotional reactions and frustration outcomes. Spector (1978) highlights a number of potential sources of frustration, including the frustrating nature of the work itself and conditions arising from the work context. Technological advances and the frustration context of the COVID-19 pandemic (see Bessière et al., 2006) over time exposes employees to information overload, frequent interruptions, multitasking (Galanti et al., 2021; Tarafdar et al., 2010), fear of the unknown, and increased stress from health risks. Consistent with the organizational frustration model, we argue that in such circumstances, employees are more likely to experience a sense of frustration—a negative emotional response resulting from obstacles or interruptions (i.e., frustration; Fox and Spector, 1999)—as a result of the techno-invasion, as one of the causative agents of technostress. Namely, feeling of frustration occurs when there is an inhibiting condition (such as those the COVID-19 pandemic posed) that obstructs realizing a goal (Lazar et al., 2006). We further argue that the frustration resulting from the techno-invasion limits employees’ ability to express their talent, creativity and to have a sense of achievement. In other words, such frustration may lead to diminished perceptions of meaningful work (i.e., negative influence on the “expressing ful potential” dimension of meaningful work). To mitigate the negative impact of frustration on meaningful work, employees need to make sense of this particular event or occurrence (Park and George, 2013) and set a goal to make their work meaningful.

To achieve the goal, employees must find proper sources of meaning that motivates their engagement and agency toward the goal (Schnell, 2009). Lips-Wiersma and Morris’s holistic model of meaningful work (2009) proposes four such sources of meaning (i.e., “developing and becoming self,” “unity with others,” “serving others,” and “expressing self”). Building on these, we argue that frustrated employees are more likely to achieve their goal of experiencing higher levels of meaningful work if they recognize their work makes a difference and meets the needs of others. Specifically, consistent with the existing studies showing that CSR contributes to meaningful work as a source of meaning (Bauman and Skitka, 2012; Glavas and Kelley, 2014), we argue that CSR perception moderates the relationship between frustration (i.e., frustration emotional response), caused by techno-invasion (i.e., frustration source), and meaningful work (i.e., frustration outcome). CSR extends the notion of work beyond one’s workplace and organization, beyond an exclusively profit-oriented perspective, and thus serves as an ideal channel for frustrated employees to find meaning through work (Aguinis and Glavas, 2019).

### Techno-invasion and meaningful work

Work fulfills our need for survival, relatedness, self-development, and self-efficacy (Blustein, 2008), and as such occupies a central position in the human search for meaning by serving as a primary source of purpose, belongingness, and identity (Michaelson et al., 2014; Rosso et al., 2010). Thus, meaningful work has become a topic of interest for many scholars and practitioners (Bailey et al., 2019) in various disciplines, including philosophy, ethics, organizational studies, economics, and sociology, leading to the development of various definitions of meaningful work and approaches on its study (Allan et al., 2019; Lysova et al., 2019). Early conceptualizations of meaningful work were unidimensional, emphasizing employees’ perceptions that their work is worthwhile, important, or valuable (Pratt and Ashforth, 2003). Allan and colleagues’ (2019) recent meta-analysis shows that some scholars have maintained this conceptualization while others (e.g., Lips-Wiersma and Wright, 2012; Rosso et al., 2010) have developed multidimensional conceptualizations that bring together aspects of the self (e.g., self-actualization and personal growth) with aspects of orientation toward others (e.g., helping others and contributing to the greater good).

Ciulla (2000) argues that meaningful work has an “objective” dimension (i.e., working conditions) and a “subjective” dimension (i.e., employee perceptions). While researchers in business ethics have explored the common element that all work and workplaces should have to facilitate meaningful work (i.e., emphasizing the objective dimension of meaningful work), scholars in organizational studies have focused their attention on examining what makes a certain task or job meaningful to a particular employee in a specific workplace (i.e., emphasizing the subjective dimension of meaningful work; Michaelson et al., 2014). However, some scholars argue that meaningful work is not associated only with specific tasks, but must also be interpreted and constructed in circumstances that may offer impoverished opportunities for meaning (Bailey et al., 2019). Contemporary workplaces increasingly encompass a strong digital dimension, which importantly shapes and constrains employee perceptions, responses, and behaviors, including those in the quest of meaning-making, and ultimately has the potential to impact salient individual and organizational outcomes.

Despite technology’s generally positive consequences, digitalization does not always necessarily produce positive outcomes (Wood et al., 2019). Indeed, the autonomy and flexibility that comes with digital work may be attractive and could help in the quest of meaning-making, but research shows that freedom and choice come with negative consequences, such as work overload and distress (Butler and Stoyanova Russell, 2018). Indicators reveal that in times of digitalization (Rutkowski and Saunders, 2018; Turel et al., 2011), life satisfaction, happiness, and interpersonal trust are declining while people are working more than ever before (Eurofound & ILO, 2017). The COVID-19 emergency has further exacerbated this (DeFilippis et al., 2020) with digitalization and technology encroaching more upon individuals at work and beyond.

Technostress encompasses the stress employees experience as a result of the potential of information technology, with the techno-invasion dimension referring to being constantly connected and thereby invading the employee’s personal life (Tarafdar et al., 2007). The COVID-19 context further facilitates the frustration response that occurs when the encroaching digitalization and its intrusion into individuals’ work and lives, that is, techno-invasion (Tarafdar and Stich, 2018), is too severe. Individuals who are too encroached with IT may feel burdened by technology and have difficulty coping with these digital demands. Digitalization has encouraged the so-called “always on” workplace culture, characterized by 24/7 access to information and connectedness. Receiving, checking, and responding to work-related emails, calls, and other messages many times during the day and often after office hours has become routine for many employees, and has been further aggravated during the COVID-19 emergency. Such techno-invasion has been shown to paradoxically decrease work productivity (Turel and Serenko, 2010). Moreover, it increases the work–life imbalance (Derks et al., 2015) and leads to various health problems, such as addiction, anxiety, and insomnia stress (Jenaro et al., 2007), as well as focus distraction (Rosen et al., 2013).

In line with these arguments and Lips-Wiersma and Morris’s (2009) holistic model, we argue that employees experiencing a techno-invasion may perceive and recognize their work as less meaningful in terms of fulfilling their potential for three distinct but interrelated reasons. First, employees who are highly techno-invaded are likely to be scattered across many different work activities, with plenty of task switching and a perception that their work does not constitute a coherent whole (Durward and Blohm, 2017). In this case, employees are more likely to receive distractions (e.g., additional tasks and requests, formal and informal communication) that require their responses and prevent them from focusing on a coherent task (Rosen et al., 2013).

Second, techno-invasion can result in technology invading not only an individual’s professional life, but also their personal life. Techno-invasion likely causes individuals to spend additional time dealing with the technology, with additional tasks and issues stemming from it. This can lead to work additionally invading their lives, resulting in reduced levels of their work–life balance (Raišienė and Jonušauskas, 2013). Indeed, meaningful work is strongly based on how individuals are able to achieve a balance between their work and non-work lives. Munn (2013) empirically demonstrated that work–life balance increases employees’ perceptions of meaningful work. In contrast, the study showed that when work–life conflict increases (i.e., when work and family/life interfere with each other and employees feel that they are inadequately fulfilling one or both of their roles), employees tend to find less meaning in their work.

Third, techno-invasion likely generates negative perceptions about work. Individuals who are heavily technologically invaded tend to equate their work with the use of technology, leading to negative perceptions about their work and low job satisfaction (Suh and Lee, 2017). Negative associations that individuals develop about their work are in turn likely to diminish their perceptions of how meaningful their work is (Rothausen and Henderson, 2019). Thus, we propose:

*H1*: Techno-invasion negatively affects meaningful work.

### The mediating role of frustration in the relationship between techno-invasion and meaningful work

Technological invasion increases digital workers’ information technology overload, leading them to feel overwhelmed and unable to cope with all the demands and invasions that digitalization places on them (Shu et al., 2011). Consistent with the Fox–Spector model of organizational frustration, we argue that this likely leads to feeling frustrated. The model of organizational frustration, which builds on the general model of frustration, specifies various sources of either mild or severe frustration (Britt and Janus, 1940; Spector, 1978; Stäcker, 1977), and such a technological invasion may accordingly act as one of these important frustration sources. Existing research shows that working faster and for longer hours, as well as being in an “always on” work culture, manifested in the constant monitoring of work-related information via digital means (e.g., email and social media), causes anxiety, insomnia, and inefficiency (Derks et al., 2015; Salanova et al., 2010). Individuals may become frustrated owing to digital encroachment, working on many different tasks and being constantly thrown off their work owing to additional information being communicated. As a result, they frequently switch among tasks, losing valuable time and becoming even more frustrated. Furthermore, the fact that techno-invasion is throwing individuals out of their work–life balance and encroaching not only their professional lives, but also their personal lives, likely further contributes to their emotional response of frustration.

In a digitally invasive setting, work has been shown to be fragmented, with lowered perceived significance (i.e., not seeing the positive impact of one’s work on others; Nemkova et al., 2019). This fragmentation and its resulting frustration can in turn undermine the experienced meaningful work (Nemkova et al., 2019; Sanchez et al., 2015). Meaningful work does not reflect a stable state (Bailey and Madden, 2017). Rather, individuals have many episodic experiences at work that are meaningful or meaningless, which they integrate into a belief system about how meaningful their work is overall. Techno-invasion-induced feelings of frustration might result in perceiving their work as meaningless or even worthless (May et al., 2004). Therefore, we propose:

*H2*: Frustration mediates the relationship between techno-invasion and meaningful work.

### The moderating role of corporate social responsibility

CSR is broadly defined as “context-specific organizational actions and policies that take into account stakeholders’ expectations and the triple bottom line of economic, social, and environmental performance” (Aguinis, 2011: 858). Recent CSR research has highlighted the importance of examining the micro-level perspective of CSR (El Akremi et al., 2018; Jones et al., 2019; Rupp and Mallory, 2015). Micro-level CSR is defined as “the study of the effects and experiences of CSR (however it is defined) on individuals (in any stakeholder group) as examined at the individual level of analysis” (Rupp and Mallory, 2015: 216). Because employees are the ones who plan, advocate, participate in, and witness CSR, scholars have begun to investigate how CSR affects employee attitudes and behaviors (Jones et al., 2017; Rupp and Mallory, 2015). Perceived CSR refers to the degree to which employees perceive their employer’s support of CSR-related activities (Choi and Yu, 2014). Because CSR-related activities are defined as a long-term and stable corporate policy in line with stakeholders’ values and resulting expectations (Žukauskas et al., 2018), we propose that CSR perceptions remain relatively stable over time.

Recent literature reviews focusing on the micro-level CSR literature have revealed that employee perceptions of CSR are associated with a number of positive consequences, including increased employee engagement, organizational citizenship behaviors, improved employee relations, and job satisfaction (see Rupp and Mallory, 2015). However, our understanding of the relationship between CSR and employee outcome relationships remains limited; thus, further research is needed to answer the questions of why, how, and when CSR has an effect on employees (Glavas, 2016).

Building on Lips-Wiersma and Morris’s (2009) holistic model of meaningful work and existing research suggesting that employees’ perceptions of CSR can facilitate meaningful work (Michaelson et al., 2014), we argue that the perception of CSR can serve as a counterbalance to frustration sources and thus reduce the negative impact of frustration techno-invasion causes on meaningful work. Rosso and colleagues (2010) argue that one way employees find meaning is by contributing to the common good or CSR. This extends the notion of work beyond one’s job and organization, and beyond an exclusively profit-oriented perspective, thus providing an ideal channel for individuals to counterbalance frustration sources and find meaning in their work (Aguinis and Glavas, 2019). Lysova and colleagues’ (2019) recent multilevel review has shown that CSR contributes to meaningful work because (a) it signals that organizations have an ethical approach toward their stakeholders, which makes employees perceive and feel a sense of pride of and identification with the organization (e.g., Glavas and Kelley, 2014); and (b) by making employees feel they are part of an effort that helps improve others’ well-being, CSR satisfies employees’ need for a meaningful existence (e.g., Bauman and Skitka, 2012).

In organizations where CSR is integrated into the organization’s strategy, routines, and operations, employees are more likely to experience meaningfulness *in* work, which arises from their own work role, and *at* work, which arises from being a part of something bigger (Aguinis and Glavas, 2019; Pratt and Ashforth, 2003). Pratt and Ashforth (2003) further argue that CSR practices, such as promoting the organization’s goals, values, and beliefs and the changing the nature of the relationship between members, can foster meaningful work. CSR can be particularly beneficial when used as a means for employees to bring meaning and their whole selves to work (Glavas and Kelley, 2014), and can provide an opportunity to re-engage individuals facing work fatigue, boredom, or even career stagnation (Aguinis and Glavas, 2019). Further, we could expect the same to be the case when frustration sources accumulating over time lead employees to an emotional response of frustration.

Therefore, we argue that employees who perceive high levels of CSR believe they are part of something bigger and can make a significant contribution to others, thereby perceiving their work as more meaningful even when they are annoyed and irritated (i.e., frustrated). CSR emphasizes the importance of an employee’s actions beyond the specific task, job, and organization, and therefore can help employees gain an understanding that their potential dissatisfaction and disappointment (i.e., frustration) serves for a bigger cause, thereby mitigating frustration’s negative effects on meaningful work. We therefore propose:

*H3*: CSR perception moderates the second stage of the indirect relationship between techno-invasion and meaningful work via frustration in such a way that this relationship is less negative for individuals with a higher CSR perception compared with individuals with a low CSR perception.

## Methods

### Sample and procedure

Through an agency specialized in data collection on work-related phenomena, we collected data from 198 working professionals across different industries with a four-wave longitudinal online survey. To match their responses across time waves, individual identification numbers were assigned to ensure participants’ anonymity. The data were collected before, during, and after the first wave of the officially declared COVID-19 pandemic in Slovenia. To describe the data collection content, Table 1 briefly summarizes the COVID-19-related situation in Slovenia in 2020 during each wave of data collection. The full sample size of participants who started with the first wave is 200; however, only 198 fully responded to all four waves of data collection, with two dropping out after the first wave. We have thus only used full respondents' data for our analyses.

**Table 1.** COVID-19-related situation in Slovenia in 2020 during each wave of data collection.

Time	Month	Situation
Time 1	Late February	Before the epidemic; preparation of potential measures
Time 2	Mid-March	The epidemic was officially declared in Slovenia; the first phase of the infection spread; a number of measures were adopted
Time 3	Mid-April	The peak of the first wave of the epidemic, the most restrictive measures were adopted
Time 4	Late May	The official end of the COVID-19 epidemic; the lockdown measures were gradually erased

The sample consisted of 46% of respondents working in public companies, 50% in private companies, and the remaining respondents working in joint ventures. Of the respondents, 10.6% were working in micro-companies with up to nine employees, 19.7% in small companies with up to 49 employees, 24.7% in medium-sized companies with up to 249 employees, and 44.9% working in large companies with 250 employees or more. Respondents operated mainly in the following industries: education, culture, and sport (13.1%); administration (12.6%); production (12.6%); health (9.6%); and sales (8.1%). Respondents were, on average, 46 years old, had 22 years of work experience, 49.5% were female, and on average, had 0.6 children. Among the respondents, 42.4% had a high school diploma, and 55% had at least an undergraduate diploma. In all, 30% performed managerial duties, and on average, worked 41.8 hours per week.

### Measures

All the focal variables were self-reported and all, except CSR, measured in all four waves. We assumed that the CSR perception was stable and would not change rapidly in the short time, thus we measured it in the first wave.

***Techno-invasion*** was assessed with three items from Shu et al.’s (2011) scale that measures technostress technology invading personal life causes. A five-point Likert scale was used with the anchors “5 = strongly agree” and “1 = strongly disagree.” Representative items include: “I have to be in touch with my work even during my vacation due to this technology,” and “I feel my personal life is being invaded by this technology” (α_t1_ = .87, α_t2_ = .86, α_t3_ = .86, α_t4_ = .89, α_cumulative_ = .87).

***Frustration*** was measured with the following item from Peters et al.’s (1980) scale: “Overall, I experienced very little frustration at work” (reverse scored). The responses ranged from “1 = strongly disagree” to “5 = strongly agree.”

***Meaningful work*** was measured with three items from Lips-Wiersma and Wright’s (2012) scale that represents expressing the full potential dimension of meaningful work. A five-point Likert scale was used with the anchors “5 = never” and “1 = very.” Representative items include: “I make a difference that matters to others,” and “I am excited by the available opportunities for me” (α_t1_ = .78, α_t2_ = .86, α_t3_ = .80, α_t4_ = .84, α_cumulative_ = .82).

***Perceived CSR*** was measured with five items Glavas and Kelley (2014) proposed. The scale covers an organization’s social and environmental responsibilities. Examples of items include: “Contributing to the well-being of the community is a high priority at my organization,” and “My organization achieves its goals while staying focused on its impact on the environment.” The responses ranged from “1 = strongly disagree” and “5 = strongly agree” (α = .84).

***Gender*** and ***age*** were measured in the first wave and incorporated in the model as individual-level control variables.

## Results

### Descriptive statistics

Table 2 shows means, standard deviation, correlations, and reliability coefficients for the key study variables. Based on Cronbach’s alpha coefficients, all measurement scales were internally consistent. They all exceeded the 0.70 criterion established in the literature (Hair et al., 1998). We first observed the factor structure of the focal variables and thus conducted a multilevel confirmatory factor analysis (MCFA) using Mplus 8.3 software (Muthén and Muthén, 1998–2012). The expected four-factor solution (techno-invasion, frustration, meaningful work, and perceived CSR) displayed adequate fit with the data (χ^2^(61) = 128.362, *p* < .01, Comparative Fit Index (CFI) = .96, Tucker Lewis Index (TLI) = .94, Root Mean Square Error of Approximation (RMSEA) = .04, Standardized Root Mean Square Residual (SRMR)_within_ = .03, SRMR_between_ = 0.05). The standardized factor loadings ranged from .64–.78 for the techno-invasion items, .52–.59 for the meaningful work, and .56–.90 for the perceived CSR items.

**Table 2.** Descriptive statistics and correlations.

	Between level	Mean	SD	1	2	3
1	Corporate social responsibility	3.42	.96	(.84)		
2	Gender	1.51	.50	.05	-	
3	Age (in years)	46.41	9.82	−.09^*^	.09^*^	-

	Within level	Mean	SD	1	2	3	4	5	6	7	8	9	10	11	12
1	Techno-invasion at t1	2.30	1.17	(.87)											
2	Techno-invasion at t2	2.42	1.14	.60^**^	(.86)										
3	Techno-invasion at t3	2.32	1.16	.53^**^	.72^**^	(.86)									
4	Techno-invasion at t4	2.34	1.18	.55^**^	.66^**^	.73^**^	(.89)								
5	Frustration at t1	2.69	1.25	.23^**^	.11	.12	.12	-							
6	Frustration at t2	2.63	1.25	.23^**^	.20^**^	.19^**^	.17^*^	.61^**^	-						
7	Frustration at t3	2.66	1.28	.16^*^	.17^*^	.24^**^	.22^**^	.45^**^	.51^**^	-					
8	Frustration at t4	2.76	1.23	.24^**^	.22^**^	.33^**^	.33^**^	.54^**^	.51^**^	.56^**^	-				
9	Meaningful work (expressing full potential) at t1	3.42	.88	−.05	−.03	−.00	−.06	−.43^**^	−.39^**^	−.44^**^	−.25^**^	(.78)			
10	Meaningful work (expressing full potential) at t2	3.36	.92	−.02	.01	.06	.04	−.35^**^	−.43^**^	−.44^**^	−.32^**^	.70^**^	(.86)		
11	Meaningful work (expressing full potential) at t3	3.40	.83	−.02	−.02	−.07	−.05	−.38^**^	−.41^**^	−.50^**^	−.38^**^	.63^**^	.68^**^	(.80)	
12	Meaningful work (expressing full potential) at t4	3.37	.86	−.11	−.07	−.09	−.07	−.44^**^	−.47^**^	−.43^**^	−.42^**^	.69^**^	.67^**^	.68^**^	(.84)

** Correlation is significant at the 0.01 level (2-tailed). * Correlation is significant at the 0.05 level (2-tailed). For gender, 1 = male, 2 = female. Reliabilities (coefficient alphas) are on the diagonal in parentheses.

As we have time-varying variables (techno-invasion, meaningful work, and frustration), we also checked for measurement invariance, to establish that participants across all groups interpret the individual questions, as well as the underlying latent factor, in the same way. Multiple CFAs were conducted for the time-varying construct of our model. CFA at time 1 (χ2 =31.80; *p =* 0.00; CFI = 0.96; TLI = 0.95; RMSEA = 0.08), time 2 (χ2 = 35.58; *p =* 0.00; CFI = 0.97; TLI = 0.95; RMSEA = 0.09), time 3 (χ2 = 42.34; *p* = 0.00; CFI = 0.95; TLI = 0.93; RMSEA = 0.10), time 4 (χ2 = 32.14; *p* = 0.00; CFI = 0.97; TLI = 0.96; RMSEA = 0.08), and a cumulative model (χ2 = 62.72; *p* = 0.41; CFI = 0.99; TLI = 0.99; RMSEA = 0.01) were thus conducted separately. Factor loadings for all constructs were significant and above .60. The results above show that the models of time-varying variables split by the time demonstrate a configural invariance.

Next, we move to metric invariance. The factor variance and mean were fixed to 1 and 0, respectively. The constraint of the first item for each factor was released so that the factor loadings and intercepts can be compared across groups (van de Schoot et al., 2012). The chi-square test (Δχ2 =12.53; *p* = 0.40) is showing invariance between groups; this is also reinforced by the CFI difference, which is less or equal or less than 0.01 (ΔCFI = 0.00) (Putnick and Bornstein, 2016). Next, we checked for scalar invariance. The factor mean and variance were fixed to 0 and 1, respectively, and all residual variances were permitted to differ across time (van de Schoot et al., 2012). The results show that compared to the metric invariance model (Δχ2 = 29.72; *p* = 0.01), the scalar model is not showing scalar invariance; we thus have scalar noninvariance. This suggests that noninvariance of the factor intercept for techno-invasion, frustration, and meaningful work (thus the scores between points) change, but this increase is not related to the change over time of the focal construct itself. Following the suggestions of Putnick and Bornstein (2016), we tried to identify the reasons behind such noninvariance. We did this by constraining the intercepts to be equal across time points and doing this for each factor separately. Techno-invasion has a significant chi-square suggesting noninvariance, meaningful work was invariant, and frustration was also nonvariant. As scalar invariance was not supported, we did not check for other steps, such as residual invariance, correlations, or means (Putnick and Bornstein, 2016; van de Schoot et al., 2012).

### Hypotheses testing

Our dataset consisted of two hierarchically nested levels: observations spanning four time points (Level 1 *n* = 792) were nested into individuals (Level 2 *n* = 198). To check this assumption—if the data are time dependent—we conducted two analyses: general linear modeling (GLM) and latent state-trait (LST) analysis in Mplus (Muthén et al., 2017). Both analyses suggested there were no significant differences in the scores for techno-invasion (GLM; F[2.375,538.828] = 1.074, NS), LST; between 48 and 81% of true individual differences in techno-invasion can be explained by individual dispositions and 52 to 19% by time), frustration (GLM; F(2.934,577.927) = .881, NS), LST; between 47 and 58% of true individual differences in frustration can be explained by individual dispositions and 53 to 42 % by time), and meaningful work (GLM; F(3,591) = .576, NS), LST; and between 71 and 86% of true individual differences in frustration can be explained by individual dispositions and 29 to 14 % by time) over time. Overall, the results of the above checks show that some key variables are not changing over time. For robustness, as another indication of variance partitioning, we also calculated the intraclass correlations (ICCs) of all the constructs that were measured across time using Biemann et al.’s (2012) Excel template. For techno-invasion, ICC(1) was .24, and ICC(2) was .64 (*F* = 2.81, *p* < .01). For frustration, ICC(1) was .32, and ICC(2) was .73 (*F* = 3.69, *p* < .01). For meaningful work (expressing full potential), ICC(1) was .22, and ICC(2) was .62 (*F* = 2.62, *p* < .01).

While there are multiple techniques available to analyze longitudinal data, we decided to apply a multilevel modeling technique to test our hypotheses, as deemed to be superior (Bell et al., 2019; Hanchane and Mostafa, 2012). For example, the authors suggest that multilevel modeling can treat uneven time intervals (as in our case, see Table 1) or model individual-level variables over time for each participant (rather than simply averages; see Kwok et al., 2008, for the list of all potential benefits). Thus, we used hierarchical linear modeling (random intercepts with fixed slopes) to test our model using multilevel structural equation modelling (SEM) in Mplus 8.3. Such an approach allows simultaneous estimation (while applying full maximum likelihood principles) of all the model’s parameters. Following the suggestions of Preacher et al. (2010), as we used a multilevel SEM, we did not center the variables prior to the analysis.

We report the results in Table 3. At the within level, the direct relationship between techno-invasion and meaningful work was negative and nonsignificant (γ = −.02, SE = .03, *p =* .56), while at the between level, the direct relationship was positive and significant (γ = .14, SE = .05, *p* < .01), rejecting Hypothesis 1. Techno-invasion was positively related to frustration at the both within-individuals (γ = .14, SE = .04, *p* < .00) and between-individuals (γ = .26, SE = .06, *p* < .00) levels. Further, frustration was negatively related to meaningful work at both the within-individuals (γ = −.10, SE = .03, *p* < .00) and between-individuals (γ = −.48, SE = .07, *p* < .00) levels.

**Table 3.** Results of multilevel analyses using Mplus.

Moderated-mediation model	Predicting frustration = mediating variable	Predicting meaningful work = outcome variable
***Within-effects (across time)***		
Techno-invasion	.14^**^ (.04)	−.02 (.03)
Frustration		−.10^*^ (.03)
***Between-effects (between-individuals)***		
Techno-invasion	.26^**^ (.06)	.14^**^ (.05)
Frustration		−.49^**^ (.07)
Interaction effect: CSR × Frustration		.15^**^ (.06)
Gender		−.05 (.07)
Age		−.00 (.00)
Model fit (DIC)	7327.50	
Within-indirect effect of frustration	−.01^*^; LLCI = −-.03; ULCI = −.01	
Between-indirect effect of frustration	−.13^**^; LLCI = −-.20; ULCI = −.06	
Between-indirect effect of frustration at low level of CSR	−.17^**^; LLCI = −.26; ULCI = −.08	
Between-indirect effect of frustration at medium level of CSR	−.13^**^; LLCI = −.20; ULCI = −.06	
Between-indirect effect of frustration at high level of CSR	−.09^*^; LLCI = −.16; ULCI = −.03	

N = 792 observations (Level 1) nested into 198 individuals (Level 2). ^*^p < .05; ^**^p < .01; ^†^*p* < .10. LLCI = lower-level confidence interval; ULCI = upper-level confidence interval.

For the next part of the analysis, confidence intervals have been calculated by the Bayesian estimator in Mplus with 20.000 interactions (Yuan and MacKinnon, 2009). In support of Hypothesis 2, the indirect relationship of frustration in the relationship between techno-invasion and meaningful work was significant at both the within (indirect effect = −.01, *p* < .05, LLCI = −.03, ULCI = −.01) and between (indirect effect = −.13, *p* < .00, LLCI = −.20, ULCI = −.06) levels. Turning to the full moderated-mediation model (Hypothesis 3), all indirect effects of the moderated-mediation were only calculated at the between level, as CSR was a level-2 construct. All the indirect effects were negative and significant (low level of CSR (−1SD), indirect effect = −.17, *p* <.00, LLCI = −.26, ULCI = −.08; medium level (mean) of CSR, indirect effect = −.13, *p* <.00, LLCI = −.20, ULCI = −.06; high level of CSR (+1SD), indirect effect = −.09, *p* <.05, LLCI = −.16, ULCI = −.03). Taken together, these results provide support for Hypothesis 3; the higher the CSR, the less negative the indirect relationship between techno-invasion and meaningful work, as mediated by frustration.^2^

## Discussion

The results of our longitudinal (four-wave) study of professionals exposed to different levels of technology use and techno-invasion before, during, and after the first wave of the COVID-19 emergency supported our proposed moderated-mediation model. Techno-invasion, as one of the causative agents of technostress, did not directly negatively influence meaningful work, but it did contribute to higher levels of frustration, indicating crucial potential downsides of the (over)use of IT in contemporary organizations. Furthermore, in certain conditions, techno-invasion reduces perceptions of meaningful work through frustration, a short-term negative outcome. As technostress is a common phenomenon in the digital context, this finding is highly relevant for understanding how the digital work environment shapes the experience of meaningful work and thus influences future meaningful work experiences. Moreover, the results show that the higher the level of CSR, the less negative the indirect relationship between techno-invasion and meaningful work, as mediated by frustration.

### Theoretical contributions

This study advances the literature on meaningful work and organizational frustration in several ways. First, our study extends previous research on meaningful work by integrating Lips-Wiersma and Morris's (2009) holistic model of meaningful work and the organizational frustration model (Fox and Spector, 1999; Spector, 1978) into a novel, comprehensive theoretical model that explains how the constraints and tensions of a context influence individuals' recognition of meaningful work. Although scholars have established that context influences the degree to which an individual can find meaningful work (Lysova et al., 2019), they have paid scant theoretical and empirical attention to the role of digitalization and crisis context in shaping employees’ experiences of meaningful work. As digitalization and high-profile events are continuously changing the nature of work, thus shaping the future of work through their impact on meaningful work, our study extends the existing literature on meaningful work by deepening our understanding of how digitalization and crisis (i.e., COVID-19 pandemic) context affect employees’ perceptions of meaningful work.

Specifically, following Lips-Wiersma and Morris's (2009) holistic model of meaningful work, we argue that stressful events arising from the context of digitalization context and the COVID-19 pandemic can trigger a crisis of meaning (i.e., evaluating life as frustratingly empty and lacking meaning). Combining this logic with the organizational frustration model (Fox and Spector, 1999; Spector, 1978), we propose that techno-invasion, arising from the digitalization context, induces a frustration emotional response and thereby reducing meaningful work. Although the literature suggests that, owing to different reasons and mechanisms, digitalization can either positively or negatively affect meaningful work (Lent, 2018; Smids et al., 2020), empirical studies rigorously examining these effects remain limited. Our study thus adds to a growing conversation about the meaningful work by theoretically and empirically investigating how digitalization and the COVID-19 context affect employees’ perceptions of meaningful work and what are the mechanisms to cultivate feelings of meaningful work under stressful conditions. Consistent with the holistic model of meaningful work that emphasizes service to others as a source of meaningful work, we propose CSR perception as an important mechanism that mitigates the negative effects of frustration (i.e., the emotional response) techno-invasion causes (i.e., frustration sources) and thereby facilitates the experience of meaningful work (i.e., frustration outcome) in the specific context (i.e., digitalization and COVID-19 pandemic). A recent review highlights the need for more empirical research exploring the positive and negative factors that shape experiences of meaningful work (Lysova et al., 2019). Therefore, this study adds to the literature on meaningful work by theoretically and empirically examining a unique set of factors (i.e., techno-invasion, frustration, CSR perceptions) that influence meaningful work. In addition, it is important to emphasize that while we proposed and empirically investigated the negative effects of context (i.e., digitization in the COVID-19 pandemic) on meaningful work, our comprehensive model is also appropriate for investigating the positive effects of context on the meaning-making process.

By outlining a comprehensive theoretical framework for how context shapes the meaning-making process, we respond to calls for examining of how interactions between various individual, organizational, and social factors contribute to the experience of meaningful work (Lysova et al., 2019) and how a broader context shapes that experience (Bailey et al., 2019). Specifically, we provide empirical support on how factors arising from the digital context (i.e., technostress and frustration) interact with a potentially mitigating factor related to a source of meaning (i.e., CSR) to influence meaningful work. By examining novel negative consequences of the techno-invasion, we also contribute to technostress research, responding to the call to explore the negative aspects (i.e., the “dark side”) of employees’ digitalization experiences (Wood et al., 2019). However, contrary to expectations, our results suggest that techno-invasion can serve as a source of meaningful work in a crisis context. In line with the existing literature, which mainly focuses on examining the negative effects of techno-invasion on employees’ work and non-work experiences (e.g., Tarafdar et al., 2010; Wu et al., 2020), we proposed that the direct relationship between techno-invasion and meaningful work is negative. However, the results suggest that the direct effect of techno-invasion on employees’ perceptions of meaningful work in crisis contexts, such as COVID-19, is actually positive. Diller and colleagues (2016) argue that techno-invasion enhances both positive and negative stress responses, depending on particular boundary conditions. For example, Wu and colleagues (2020) found that employee computer self-efficacy and perceived organizational support can significantly mitigate the negative consequences of techno-invasion. Because our data were collected during the COVID-19 pandemic when many employees performed their work remotely (Molino et al., 2020), many organizations were forced to pay special attention to employee support to maintain continuous implementation of business processes. In addition, employees who had to work during the first wave of the pandemic possibly received reassurance that their work, although intruding on their personal life, was important and worth doing, serving as a source of meaning, which in turn increased their perception of how meaningful work actually it was. However, our theorization and results also suggest a negative indirect effect of techno-invasion on meaningful work through increased frustration. If techno-invasion leads to frustration, the degree to which employees find their work meaningful will decrease.

Moreover, in line with Lips-Wiersma and Morris's (2009) holistic model of meaningful work, we also highlight CSR’s crucial importance as a source of meaning, counterbalancing sources of frustration and promoting meaningful work. Thereby, our study responds to the call to investigate conditions under which CSR can lead to win–win outcomes of business value and employee well-being simultaneously (Aguinis and Glavas, 2019). By examining the moderating role of CSR perception in the indirect relationship between techno-invasion and meaningful work, which frustration mediates, our study contributes to the discourse on CSR’s positive impact related to meaningful work. Existing evidence suggests that CSR is particularly beneficial when used as a means for employees to bring both more meaning and their whole selves to work (Glavas and Kelley, 2014), and provides an opportunity to re-engage individuals who are in a challenging situation (Aguinis and Glavas, 2019). Consistent with the existing evidence, our study suggests that CSR perception can mitigate the negative consequences of frustration techno-invasion causes, highlighting CSR’s critical importance in promoting meaningful work in the digital context.

Second, by exploring how situational events arising from digitalization and the COVID-19 context affect the organizational frustration model’s three elements, our study also contributes to the literature on organizational frustration. Building on the idea that situational factors are related to specific frustration source (Bessière et al., 2006), we proposed a novel source of frustration (i.e., techno-invasion) and explained how it is linked to the frustration emotional response (i.e., frustration) and the novel frustration outcome (i.e., meaningful work) as the Fox–Spector model of organizational frustration predicts. We found empirical evidence that the techno-invasion contributed to higher levels of frustration, thereby advancing the existing debate in the literature on organizational frustration and meaningful work, which has mainly examined how of meaningful work can mitigate the negative effects of frustration (e.g., Ugwu and Onyishi, 2018). Our study shows that certain events can affect the organizational frustration model’s three elements and thus meaningful work. One of the novelties of this study is also to shed light on how CSR perceptions, which are influenced by corporate CSR policies, can mitigate the negative effects of the frustration emotional response resulting from the frustration source on the frustration outcome.

Third, our study also makes an empirical contribution by testing the moderated-mediation model, which posits frustration as a mediator of techno- invasion effects on meaningful work, with CSR perceptions as a moderator of such effects using a four-wave longitudinal study conducted during the first wave of the COVID-19 pandemic. As a global phenomenon with severe global consequences across all aspects of work and life, COVID-19 further exacerbated some negative consequences of digitalization and acted as an important contingency interfering and constraining workers’ experiences of frustration (Bessière et al., 2006). Thus, we advance research on the experience of meaningful work under these unique and stressful conditions.

### Practical implications

Our findings have important practical implications for creating work environments that aim to maximize employees’ perceptions of meaningful work, particularly with regard to administering appropriate levels of technological invasion. There is ongoing public and professional debate about the negative impact of techno-invasion on individuals, which may also negatively affect organizationally relevant outcomes. Our study provides the rare empirical evidence that techno-invasion can have both positive and negative impact on meaningful work. Specifically, our results show that in times of crisis (e.g. COVID-19 pandemic), when employees are aware of the importance of their work for the survival of the organization, the techno-invasion can positively influence their perception of meaningful work. However, our results also show that techno-invasion can also lead to frustration, decreasing employees' perception of meaningful work. Therefore, managers and organizations should carefully examine their employees' attitudes toward the use of technology, keep track of their workloads and overloads, and monitor whether employees perceive technological invasion as a challenge that enables them to express their full potential or as a source of frustration. To avoid employees feeling frustrated and thereby believing their work is less meaningful, organizations should keep an eye on digital intrusion into employees’ lives and pay attention to how much work is assigned to them through technological means, as well as when that work is administered. Moreover, frustration occurs when employees feel that some inhibiting factors, such as the work environment and the structure of the working environment, including procedures and rules, prevent them from achieving their goals (Lazar et al., 2006; Spector, 1978). Therefore, to reduce the techno-invasion-induced frustration, organizations should apply rules and policies to limit after-hours work (e.g., by instituting a “no after-hours” or a “limited timeframe email” policy), so that employees can achieve their goals in both their professional and personal lives. Organizations should also keep formal expectations of employees’ availability at all times and places low (Piszczek, 2017) and encourage them to take time off from work and technology to reduce feelings of technological invasion, thereby reducing frustration.

Second, even if employees feel that technology is invading their work and lives, our findings suggest that organizations can prevent this occurrence from resulting in reduced feelings of how meaningful work they perform is by focusing on inducing higher levels of CSR perceptions. Organizations and managers should therefore carefully design and implement CSR practices, policies, and actions, as they can significantly influence employees’ CSR perceptions and thus their perception of meaningful work in a crisis context. Organizations can use human resource management practices and systems, such as training and development, to make employees aware of CSR polices (Shen and Benson, 2016). Once they become aware that their organizations give them the opportunity to positively contribute to the world, they may become re-energized and find meaningfulness in their work (Aguinis and Glavas, 2019) despite techno-invasion-induced feelings of frustration. In addition, our study shows that employees are more likely to experience their work as meaningful in crisis situations if they feel they are part of something bigger. This finding suggests that organizations should pay particular attention to promoting meaningfulness at work—a sense of meaning that comes from being part of the organization rather than from what one does. Pratt and Ashforth (2003) suggest that meaningfulness at work can be fostered through building cultures, ideologies, identities, and communities, as well as through charismatic, visionary, or transformational leadership.

Practically, our study implies that organizations should: (1) review and analyze business processes and core values to identify where values, such as solidarity, environmental awareness, or contribution to humanity, exist or could potentially exist (Asif et al., 2013) and integrate them into the corporate culture; (2) carefully develop CSR initiatives and adapt them to the particular organizational context and promote them even in difficult circumstances as they may influence employees’ CSR perceptions, thereby increasing meaningful work; and (3) promote CSR initiatives internally as well as externally, integrating them with people management and marketing strategies, and (co)create positive (employee) brand awareness (Bhattacharya et al., 2004; Jamali et al., 2015) to promote meaningfulness at work.

### Limitations and future research directions

As is true for any research, our study is not without limitations. While our longitudinal research across four points in time entails important advantages in terms of making causal claims, a possible limitation of our research design can be the exclusive use of self-reports. However, according to Fox and Spector (1999), self-reported measures capture critical features of the situation more adequately than more objective, non-intuitive measures. Because our study aimed to understand how employees view, feel, and respond to digitalized work, a self-report methodology made the most sense (Howard, 1994; Spector, 1994). Nonetheless, such research could be complemented by including additional objective measures, perhaps those of CSR, by investigating a multilevel model of organizational CSR initiatives that moderate the basic mediated model at the individual level, or expand the model to include potential impact on business performance.

In terms of our used measurement instruments, for several constructs (specifically for technostress and meaningful work), we have only captured a single dimension of otherwise multidimensional constructs, and short and even single-item scales had to be used. Such an approach might be especially useful in longitudinal research in an attempt not to overburden respondents with too long research instruments, thus enabling them to maintain concentration and focus on the content when responding (Fisher et al., 2016; Lucas and Donnellan, 2012). During the COVID-19 pandemic, when individuals faced a range of professional, personal, and health challenges that required a rapid response and the time and energy of individuals, it was even more important that we were considerate of their time and kept the survey as short as possible. Further, the selected dimensions were chosen because they were theoretically the most relevant for the research model in question. However, future research could further improve the validity and scope of our study by employing multidimensional scales and investigating whether additional dimensions of technostress and meaningful work behave differently.

In addition, we have captured techno-invasion, frustration, and meaningful work across time, while only relying on the CSR perceptions the respondents provided in a single point in time. Because our study took place across several months, it is also possible that some respondents changed jobs in this period, which would likely change their perceptions regarding their organizations’ CSR. Since we have a nationally representative quota sample (representative of age, gender, and industry), we can assume, based on our national labor statistics, that only a small number of employees included in the sample changed jobs. However, a viable research avenue would be to examine how CSR perceptions are shaped over time, which would involve longer periods included in such longitudinal research, potentially spanning across multiple years. This would also produce larger variance in all examined constructs over time. Lastly and on a related note, our preliminary checks highlighted that few constructs (especially techno-invasion and frustration) did not change over time or there was no scalar invariance. This means that comparison between time points might not be needed as there are differences in the scale meaning for some constructs is different between time points, or that the change over time is less prominent that expected. Even if some authors still compare groups/time points even without assessing scalar invariance (e.g., Dahlstrom and Nygaard, 1995) and multilevel analysis is recommended, we believe that our results should be taken with this limitation in mind.

## Notes

1 Referring to the disease caused by the “SARS-CoV-2” virus and how it has modified work-related phenomena.

2 The results with regards to tests of hypotheses also hold when using random slopes for the link between frustration and meaningful work (where the moderation of CSR is tested). As a robustness check using an alternative analytic technique and software, we also applied MLmed (Beta 2), an SPSS (v25) macro for fitting multilevel models (Hayes and and Rockwood, 2020; Rockwood and Hayes, in press). The results were very similar and consistent in terms of tests of hypotheses.
